# Examine sex differences in autism spectrum disorder in school-aged children and adolescents with fluent language

**DOI:** 10.3389/fpsyt.2023.1151596

**Published:** 2023-04-06

**Authors:** Yan Ji, Yue Ji, Hui-lin Zhu, San-mei Cheng, Xiao-bing Zou, Feng-lei Zhu

**Affiliations:** Child Developmental and Behavioral Center, The Third Affiliated Hospital of Sun Yat-sen University, Guangzhou, China

**Keywords:** autism spectrum disorders, sex difference, intelligence quotient, core symptoms, autism diagnostic observation schedule, autism diagnostic interview-revised, Wechsler intelligence scale for children-fourth edition (Chinese version)

## Abstract

There are noteworthy sex disparities in the prevalence of autism spectrum disorders (ASD), while findings regarding the sex differences in core symptoms are inconsistent. There are few relevant studies on sex differences in mainland China. This study was dedicated to a deeper understanding of the impact of sex differences on the clinical presentation of ASD with fluent language. We retrospectively studied 301 children with ASD (58 females) and utilized raw scores from the ADI-R and ADOS and the intelligence quotient (IQ) to measure symptomatology. Based on the Full-Scale IQ (FS-IQ), a binary split of average, above-average IQ (high-IQ), and below-average IQ (low IQ) occurs at 85. Across the entire sample, males and females are comparable in the FS-IQ, while males scored higher in the Perceptual Reasoning Index (PRI) (*F* = 7.812, *p* = 0.006). ADI-R did not find any statistically significant sex differences in the diagnostic cutoff score satisfaction or the raw domain scores. While a significant effect of sex on ADOS social affect domain scores was found in the total sample [*λ* = 0.970, partial *η^2^* = 0.030, *F* (3,295) = 3.019, *p* = 0.030]. Tests of between-subjects effects revealed that males scored higher than females mainly in the ADOS reciprocal social interaction subcategory (partial *η^2^* = 0.022, *F* = 6.563, *p* = 0.011). Stratified analysis revealed that the effect of sex on ADOS reciprocal social interaction subcategory scores only significant in the low-IQ children with ASD (partial *η^2^* = 0.092, *F* = 10.088, *p* = 0.002). In general, overall cognitive functioning is similar across males and females with ASD, while males have a higher perceptual reasoning ability. Females with ASD are more likely to have comorbid intellectual impairment than males, and they could require additional intervention support. Autistic children with low IQs are more likely to exhibit sex differences in their core symptoms than children with high IQs. Intelligence plays a key role in sex-based differences in the core symptoms of ASD.

## 1. Introduction

The predominant features of autism spectrum disorder (ASD), a neurodevelopmental disorder that appears in the early stages, are persistent challenges with social interaction and communication, as well as restricted and repetitive behaviors (RRB), interests, and activities (1). Although the exact cause of ASD is still unknown, it is widely acknowledged that gene–environment interactions play a role in its development. The prevalence of ASD has considerably increased in recent years. The latest estimations of the prevalence of ASD among children aged 8 in the United States showed that it was one in 44, with a male-to-female sex ratio of 4.2:1, and the prevalence of ASD in both males and females is substantially affected by their cognitive performance (2). Individualized care and treatment for children with ASD are increasingly advocated by the international community since they impose a substantial burden on the affected people and their families (3).

Gender, or sex, is a critical concept in autism research and clinical practice. Gender is a socio-cultural construct, whereas sex is a biological or physiological attribute (4). This study used the term “sex” to define how males and females differ from one another. The child’s development history, symptom observation, and clinical confirmation are the rudimentary building blocks of the ASD diagnostic process. For a precise diagnosis and a personalized intervention, it is essential to consider the sex differences in the clinical presentation of children with ASD. As the gold standard for autism diagnosis, the Autism Diagnostic Interview-Revised (ADI-R) (5) and the Autism Diagnostic Observation Schedule (ADOS) (6) (both abbreviated as “Double A”) were both used to evaluate the severity of autism symptoms. It is challenging for research workers to compare the symptom severity of children with ASD who belong to different age groups using cross-module raw scores due to obstacles that have been put in place to account for the developmental trajectories of age as well as the varied testing tasks in each module of the ADOS. The calibrated severity score (CSS), a standardized 10-point severity metric that delivers a quantitative assessment of symptom severity, was developed as a solution to this problem (7). Perhaps, it would prompt concerns about whether CSS will somehow weaken the ability of this diagnostic tool to distinguish sex differences in ASD symptoms.

It is well recognized that there are significant sex disparities in the prevalence of ASD, while the sex differences at the core phenotypic manifestations of ASD are inconsistent. A large European multi-site sample study employing gold-standard diagnostic methods found that females and males with ASD experienced comparable levels of social interaction and communication difficulties, while early childhood restricted and repetitive behaviors were less prevalent in females than in males (8). Not only that, but a recent meta-analysis that included 11 original studies also found no evidence of sex differences in social and communication function in either ASD or ADHD children (9). Additionally, it has been demonstrated that boys and girls did not differ in terms of scores on commonly used screening instruments (Modified-Checklist for Autism in Toddlers-Revised with Follow-up and Social Communication Questionnaire) (10). Since the sex difference among ASD children is not adequately captured by standard measures, there are divergent opinions on this aspect. Both the disproportionately biased “male lens” and lack of knowledge about the ASD symptoms unique to females hampered eventual referral and diagnosis (11, 12). Compared to males, females received ASD diagnoses later (13). Female individuals with ASD had significantly better social interaction and social communication skills, according to the comprehensive conclusions of a systematic review and meta-analysis of sex/gender differences, while a similar sex/gender profile has been found in non-autistic people (14). Following a previous review, autistic females may display female-gender-typical narrow interests, higher social attention, linguistic abilities, motivation for friendship, and more camouflage than autistic males (15). In addition, severity trajectory studies also establish sex differences at varying levels. Symptom severity decreases were more common during early childhood, and girls experienced greater symptom severity decreases and fewer symptom severity increases than boys (16, 17). The variability between investigations may have been partially explained by the heterogeneity of the measures or tools utilized. Additionally, studies have established that cultural issues and an individual’s age play a significant role in sex differences, which can lead to erratic and inconsistent results (10, 14).

Sex differences in ASD have gained growing research attention, yet there is a paucity of studies exploring sex differences in low-resource settings, although the majority of autistic people reside in low- and middle-income countries rather than high-income ones (18). Studies comparing male and female ASD patients had some limitations, such as small female sample sizes, a wide age range, the use of cross-version testing tools, and some studies did not consider participants’ cognitive and developmental abilities, as well as language proficiency (19–21). When it comes to measuring cognitive ability, results may vary depending on the participant’s age and the type of cognitive test employed (22, 23). Only a few studies in mainland China evaluated sexual differences in the clinical profiles of children with ASD. The clinical phenotypes of preschool children with ASD differ between boys and girls, according to research conducted previously by colleagues at our center. They discovered that girls with ASD exhibit more socio-emotional reciprocity and fewer RRBs than boys, and the types of RRBs that girls exhibit are different from those of boys (24). It has been proposed, however, that person-environment fit is a key determinant of functioning for autistic people in general, where environmental factors such as excessive social communication demands or bullying will increase disability, whereas a tolerant social environment or a suitable workplace will instead facilitate abilities (25). Due to this, findings may be inconsistent between preschool-age and school-age children. To better understand the impact of sex differences on the core symptoms of ASD, a single-center retrospective case–control study was designed to minimize the confounding effects on the outcome. In this study, the core symptoms of school-aged children and adolescents with ASD were evaluated for sex differences utilizing raw scores from the ADOS and ADI-R assessments.

## 2. Materials and methods

### 2.1. Participants

We retrospectively studied 301 children with ASD, investigating their core symptoms and looking for any sex differences. All the participants were diagnosed with ASD at the Child Developmental and Behavioral Center in the Third Affiliated Hospital of Sun Yat-sen University, from January 2019 to June 2022. Participants, which consist of 243 male and 58 female patients, were selected based on their age and language ability. In essence, the sample selected for this study is a convenience sample. We collected all eligible cases diagnosed at our center in recent years.

### 2.2. Inclusion and exclusion

#### 2.2.1. Inclusion criteria

- The chronological age ranged from 6 to 16 years old.
- Had the first diagnosis of ASD according to the fifth edition of the Diagnostic and Statistical Manual of Mental Disorders (DSM-5) (1).
- Verbally fluent: children who can be spontaneous and flexibly use sentences with multiple clauses that describe logical connections between sentences, and can talk about objects or events not immediately present (6).
- The ADI-R, WISC-IV, and ADOS (Module 3) assessments were all completed at the same time.
- Parents signed an “informed consent” at the time of the hospital visit, giving their authorization for the use of their child’s assessment data for scientific analysis.

#### 2.2.2. Exclusion criteria

Schizophrenia, co-occurring visual impairment, and hearing impairment were excluded.

### 2.3. Measures

A battery of tests, including the gold-standard ASD diagnostic assessments (ADI-R and ADOS) and the Wechsler Intelligence Scale for Children-Fourth Edition (Chinese Version) (WISC-IV) (26), were to be leveraged to measure symptomatology.

#### 2.3.1. The autism diagnostic observation schedule

The ADOS consists of four modules that are appropriate to different levels of expressive language and is a semi-structured, standardized evaluation of social interaction, communication, and restricted and repetitive behaviors (RRB) for people who are suspected to have ASD (6). Module 3 contains 13 tasks and 28 rating items, but not all ratings are used as the algorithm items of the diagnostic reference score. This module requires that participants have fluent verbal skills and the ability to talk about things that are not immediately present. Each rating item was graded from severe to moderate to typical, with severe being scored as 2, indicating “definite evidence of autism,” moderate being rated as 1, suggesting “partially but not entirely evidence of autism,” and typical being rated as 0, denoting “no evidence of abnormality related to autism.” These tasks and rating items constitute three ADOS subcategories: the communication subcategory (domain A, with a diagnostic cutoff of 3 points for autism and 2 points for ASD), the reciprocal social interaction subcategory (domain B, with a diagnostic cutoff score of 6 points for autism and 4 points for ASD), and the restricted and repetitive behavior subcategory (domain C, without the cutoff score). Domain A and Domain B constitute the social affect domain (domain A and B), and the diagnostic cutoff score is 10 points for autism and 7 points for ASD. The subject’s scores on the three ADOS domains all need to be greater than or equal to the relevant cutoff scores for autism/ASD to be diagnosed. For statistical analysis, the raw scores of the full scale and each subdomain and their relation to the cutoff scores were evaluated. The full-scale score of ADOS is the result of adding up the raw scores of each domain. The diagnostic compliance rate represents the percentage of individuals in the group whose domain scores satisfy the cutoff scores of the autism or ASD diagnostic criteria.

#### 2.3.2. The autism diagnostic interview-revised

ADI-R is a semi-structured, investigator-based interview for caregivers of children suspected of having a diagnosis of autism or pervasive developmental disorders, appropriate for children of mental ages ranging from approximately 18 months into adulthood (5). The primary clinical interview questions are divided into five sections: the opening questions, the communication questions, the social development and play questions, the repetitive and restricted behavior questions, and the general behavior problems. The caregivers’ responses are then encoded using two predominant scoring algorithms. Based on the coding methods, a score of 0 indicates “no definite behavior of the type specified,” a score of 1 represents “behavior of the type specified probably present but defining criteria not fully met,” a score of 2 indicates “definite abnormal behavior of the type described in the definition,” and a score of 3 was used to describe the extreme severity, where the highest scores indicate the highest degree of deficiency. Moreover, severity scores of 2 and 3 are both coded as 2. Under the algorithm for diagnosis, 16 items were established to measure reciprocal social interaction (domain B′), 13 items to measure communication (domain A′), and 8 items to measure RRB (domain C′). The diagnostic cutoff score for verbal subjects is 8 points in domain A′, 10 points in domain B′, and 3 points in domain C′. To be diagnosed with autism, a child must meet the diagnostic cutoff score in each of the three domains and exhibit specific aberrant behaviors that were present at or before the age of 36 months (domain D′), as reported by the caregiver or determined by the interviewer. Raw scores of the full scale or each domain and their relation to the diagnostic cutoff scores were assessed for statistical analysis. The full-scale score of ADI-R is the result of adding up the raw scores of each domain.

#### 2.3.3. The Wechsler intelligence scale for children-fourth edition-Chinese version

The Chinese version of the WISC-IV, which was revised by H. C. Zhang and his team in 2007, has the same functional properties as the original version of the WISC-IV and is good for clinical application (26, 27). The WISC-IV will output four sub-scale indices: the Verbal Comprehension Index (VCI), the Perceptual Reasoning Index (PRI), the Working Memory Index (WMI), and the Processing Speed Index (PSI), as well as a full-scale IQ (FS-IQ). All individuals were measured with the Chinese version of the WISC-IV, and their FS-IQ would be used as a basis for stratifying. Children’s capability on some scales may be too low to reliably measure, hence the FS-IQ is an estimate due to “the floor effects” in intelligence tests. Average and above-average IQs (≥85) were defined as high IQs, while below-average IQs (<85) were defined as low IQs. We IQ-stratified our analysis by separately examining the two groups. Two medical staff members reviewed the research dataset after it was extracted from the hospital’s electronic case management system.

### 2.4. Statistical analysis

Statistical analyses were conducted using IBM SPSS statistical software (version 20.0). The age difference between males and females was tested with the *t-*test. The difference in domain scores for satisfying cutoff scores of the autism or ASD diagnostic criteria was tested with the *Chi-square* test. IQ, ADI-R domain scores, and ADOS scores in the domain of social affect obeyed or nearly obeyed the normal distribution and satisfied the homogeneity of variance in Levene’s test. Sex differences were tested using multivariate analysis of covariance (MANCOVA), with sex as the fixed factor and age and FS-IQ as covariates. ADOS RRB scores were skewed, and non-parametric tests (Mann–Whitney *U* tests) were applied to compare groups. At *p* < 0.05, the difference was statistically significant.

## 3. Results

### 3.1. Individual general information and sex differences in IQ

The sample as a whole consisted of 301 participants, with 58 females, giving the male/female sex ratio of 4.19:1. In the total sample, the average age was 114.23 (30.85) months, and the average FS-IQ was 92.17 (19.24). The age difference between males and females was not statistically significant (*t* = −0.997, *p* = 0.320). Multivariate tests revealed a significant effect of sex on IQ [*λ* = 0.941, *partial η^2^* = 0.059, *F* (5,295) = 3.713, *p* = 0.003]. Tests of between-subjects effects revealed that males and females were roughly comparable in FS-IQ, VCI, WMI, and PSI (Table 1). While males scored higher than females in the PRI (*F* = 7.812, *p* = 0.006).

**Table 1:** Test of the between-subjects effect of sex on the intelligence of children with ASD.

	Males (*n* = 243) Mean (SD)	Females (*n* = 58) Mean (SD)	MANOVA			
			*F* value	*df*	*p* value	partial *η^2^*
FS-IQ	92.95 (17.98)	88.88 (23.70)	2.106	1	0.148	0.007
VCI	95.23 (20.08)	91.10 (24.30)	1.814	1	0.179	0.006
PRI	98.82 (17.93)	91.10 (22.55)	7.812	1	0.006	0.025
WMI	92.31 (16.56)	91.52 (22.00)	0.093	1	0.760	<0.001
PSI	87.40 (17.16)	85.66 (20.67)	0.446	1	0.505	0.001

FS-IQ, The Full-Scale IQ; VCI, The Verbal Comprehension Index; PRI, The Perceptual Reasoning Index; WMI, The Working Memory Index; PSI, The Processing Speed Index; MANOVA, multivariate analysis of variance.

### 3.2. Individual features of children with ASD in different IQ groups

There were 104 children with ASD (female = 24) in the group of low IQs and 197 (female = 34) in the high-IQ group (Table 2). Children in the high-IQ group were older than those in the group of low IQs (*t* = 2.160, *p* = 0.032), and the FS-IQ of the former was significantly higher (*t* = 20.695, *p* < 0.001). The male/female sex ratio was slightly higher for children in the high-IQ group, but the difference was not significant (*x^2^* = 1.481, *p* = 0.224). In the stratified analysis, males and females from the high-IQ group were roughly comparable in FS-IQ (*t* = −0.818, *p* = 0.414) and age (*t* = −0.964, *p* = 0.336), as were males and females from the low-IQ group in age (*t* = −0.013, *p* = 0.990). While males had a higher FS-IQ than females in the low-IQ group (*t* = 2.856, *p* = 0.005).

**Table 2:** Individual characteristics of children with ASD in different IQ groups.

	High IQ (*n* = 197) Mean (SD)	Low IQ (*n* = 104) Mean (SD)	*t*-test/Chi-square test			
			df	*t*/*x*^2^ value	*p* value	95% CI of the mean difference
Age (Month)	117.01 (29.69)	108.97 (32.45)	299	2.160	0.032	0.72,15.35
FS-IQ	102.87 (13.20)	71.88 (10.55)	299	20.695	<0.001	28.04,33.94
Sex ratio (M/F)	4.79:1	3.33:1	1	1.481	0.224	

High IQ: IQ ≥ 85; Low IQ: IQ < 85; FS-IQ, The Full-Scale IQ; M/F, male/female; CI, confidence interval.

### 3.3. Sex differences at the autism diagnosis compliance rate of ADI-R

Both in the total sample and the high- or low-IQ group of children with ASD, we did not find any statistically significant sex differences in the diagnosis compliance rates (Table 3).

**Table 3:** Comparisons of the autism diagnosis compliance rate in the subdomains of ADI-R between males and females with ASD.

Domain	The total sample [*n* (%)]					High IQ [*n* (%)]					Low IQ [*n* (%)]				
				Chi-square test					Chi-square test					Chi-square test	
	Male	Female	*df*	*x^2^*	*p*	Male	Female	*df*	*x^2^*	*p*	Male	Female	*df*	*x^2^*	*p*
A′	211 (86.83)	51 (87.93)	1	0.050	0.823	138 (84.66)	28 (82.35)	1	0.113	0.737	73 (91.25)	23 (95.83)	1	0.091	0.762
B′	203 (83.54)	52 (89.66)	1	1.353	0.245	132 (80.98)	30 (88.24)	1	1.013	0.314	71 (88.75)	22 (91.67)	1	0.001	0.977
C′	205 (84.36)	50 (86.21)	1	0.123	0.726	136 (83.44)	27 (79.41)	1	0.319	0.572	69 (86.25)	23 (95.83)	1	0.855	0.355
D′	234 (96.30)	55 (94.83)	1	0.264	0.607	156 (95.71)	34 (100.00)	1	1.514	0.219	78 (97.50)	21 (87.50)	1	2.145	0.143
Full Scale	176 (72.43)	42 (72.41)	1	<0.001	0.998	115 (70.55)	23 (67.65)	1	0.113	0.737	61 (76.25)	19 (79.17)	1	0.088	0.766

High IQ: IQ ≥ 85; Low IQ: IQ < 85; Domain A′, the domain of communication of ADI-R; Domain B′, the domain of social interaction abnormality of ADI-R; Domain C′, the domain of restricted and repetitive behaviors of ADI-R; Domain D′, the domain of exhibit specific aberrant behaviors that were present at or before the age of 36 months.

### 3.4. Sex differences in the autism and ASD diagnosis compliance rate of ADOS

Based on the autism or ASD cutoff scores in ADOS, we found no statistically significant sex differences in the diagnostic compliance rates both in the total sample and in the high- or low-IQ group of children (Table 4).

**Table 4:** Autism or ASD diagnosis compliance rate in ADOS.

Domain		The total sample [*n* (%)]					High IQ [*n* (%)]					Low IQ [*n* (%)]				
					Chi-square test					Chi-square test					Chi-square test	
		Male	Female	*df*	*x^2^*	*p*	Male	Female	*df*	*x^2^*	*p*	Male	Female	*df*	*x^2^*	*p*
A	ASD	206 (84.77)	45 (77.59)	1	1.746	0.186	129 (79.14)	23 (67.65)	1	2.109	0.146	77 (96.25)	22 (91.67)	1	0.142	0.706
	autism	157 (64.61)	32 (55.17)	1	1.785	0.182	90 (55.21)	15 (44.12)	1	1.392	0.238	67 (83.75)	17 (70.83)	1	1.239	0.266
B	ASD	236 (97.12)	55 (94.83)	1	0.218	0.640	156 (95.71)	31 (91.18)	1	0.442	0.506	80 (100)	24 (100)	1	<0.001	1.000
	autism	200 (82.30)	47 (81.03)	1	0.051	0.821	128 (78.53)	28 (80.56)	1	0.250	0.617	72 (90.00)	19 (79.17)	1	1.114	0.291
A + B	ASD	221 (90.95)	50 (86.21)	1	1.172	0.279	142 (87.12)	28 (82.35)	1	0.212	0.645	79 (98.75)	22 (91.67)	1	1.261	0.261
	autism	147 (60.49)	30 (51.72)	1	1.487	0.223	80 (49.08)	13 (38.24)	1	1.327	0.249	67 (79.76)	17 (70.83)	1	1.239	0.266
Full Scale	ASD	200 (82.30)	45 (77.59)	1	0.688	0.407	123 (75.46)	23 (67.65)	1	0.895	0.344	77 (96.25)	22 (91.67)	1	0.142	0.706
	autism	134 (55.14)	27 (46.55)	1	1.390	0.238	72 (44.17)	12 (35.29)	1	0.907	0.341	62 (77.50)	15 (62.50)	1	2.161	0.142

High IQ: IQ ≥ 85; Low IQ: IQ < 85; Domain A, the domain of communication of ADOS; Domain B, the domain of reciprocal social interaction of ADOS.

### 3.5. Autism diagnosis compliance rate in both ADI-R and ADOS assessment

In the total sample, the difference in autism diagnosis compliance rate between males and females was not statistically significant. According to results from Table 5, it can be calculated that, for the total sample, the vast majority of males (84.36%) and females (82.76%) met the autism diagnostic condition in at least one of the diagnostic schedules. In a similar vein, the sex difference in the autism diagnosis compliance rate was insignificant in both the high-IQ and low-IQ groups.

**Table 5:** Autism diagnosis compliance rate in both ADI-R and ADOS assessment.

	The total sample [*n* (%)]					High IQ [*n* (%)]					Low IQ [*n* (%)]				
				Chi-square test					Chi-square test					Chi-square test	
	Male	Female	*df*	*x^2^*	*p*	Male	Female	*df*	*x^2^*	*p*	Male	Female	*df*	*x^2^*	*p*
Meet both ADI-R and ADOS	105 (43.21)	21 (36.21)	2	0.948	0.623	55 (35.74)	9 (26.47)	2	0.792	0.673	50 (62.50)	12 (50.00)	2	1.418	0.492
Meet either ADI-R or ADOS	100 (41.15)	27 (46.55)				77 (47.24)	17 (50.00)				23 (28.75)	10 (41.67)			
Meet neither ADI-R nor ADOS	38 (15.64)	10 (17.24)				31 (19.02)	8 (23.53)				7 (8.75)	2 (8.33)			

High IQ: IQ ≥ 85; Low IQ: IQ < 85.

### 3.6. Sex differences among children with ASD in the domain scores of ADI-R and ADOS

In the total sample, MANCOVA (with age and FS-IQ as covariates) revealed that there was no discernible difference between males and females on the ADI-R domain scores [*λ* = 0.984, *partial η^2^* = 0.016, *F* (4,294) = 1.157, *p* = 0.330] (Table 6). Besides, no significant sex differences were found either in high-IQ children with ASD [*λ* = 0.984, *partial η^2^* = 0.016, *F* (4,190) = 0.770, *p* = 0.546] or in low-IQ children with ASD [*λ* = 0.959, *partial η^2^* = 0.041, *F* (4,98) = 1.041, *p* = 0.390]. In the ADOS RRB domain, the Mann–Whitney *U* tests did not find any significant statistical difference between males and females in the total sample (*Z* = −1.902, *p* = 0.057), or in the high-IQ (*Z* = −1.653, *p* = 0.098) or low-IQ (*Z* = −1.424, *p* = 0.154) children with ASD (Table 7). In the ADOS social affect domain, there were significant sex differences in the total sample [*λ* = 0.970, *partial η^2^* = 0.030, *F* (3,295) = 3.019, *p* = 0.030] and in the low-IQ children with ASD [*λ* = 0.878, *partial η^2^* = 0.122, *F* (3,98) = 4.555, *p* = 0.005]. While no significant effect of sex on ADOS scores was found in the high-IQ children with ASD [*λ* = 0.984, *partial η^2^* = 0.016, *F* (3,191) = 1.018, *p* = 0.386] (Table 7). In the total sample, tests of between-subjects effects revealed that males scored higher than females in the ADOS reciprocal social interaction subcategory (*partial η^2^* = 0.022, *F* = 6.563, *p* = 0.011) and in the ADOS full-scale scores (*partial η^2^* = 0.023, *F* = 7.007, *p* = 0.009). In the low-IQ children with ASD, tests of between-subjects effects revealed that males scored higher than females in the ADOS reciprocal social interaction subcategory (*partial η^2^* = 0.092, *F* = 10.088, *p* = 0.002) and in the ADOS full-scale scores (*partial η^2^* = 0.088, *F* = 9.624, *p* = 0.002).

**Table 6:** Sex differences among children with ASD in the domain scores of ADI-R.

Domain	The total sample							High IQ							Low IQ						
	Male (*n* = 243) Mean (SD)	Female (*n* = 58) Mean (SD)	MANCOVA (Wilks’ Lambda)					Male (*n* = 163) Mean (SD)	Female (*n* = 34) Mean (SD)	MANCOVA (Wilks’ Lambda)					Male (*n* = 80) Mean (SD)	Female (*n* = 24) Mean (SD)	MANCOVA (Wilks’ Lambda)				
			*λ*	*F value*	*df*	*p* value	partial *η^2^*			*λ*	*F value*	*df*	*p* value	partial *η^2^*			*λ*	*F value*	*df*	*p* value	partial *η^2^*
A′	11.59 (4.14)	12.07 (4.35)	0.984	1.157	4,294	0.330	0.016	11.18 (4.06)	10.91 (4.06)	0.984	0.770	4,190	0.546	0.016	12.41 (4.20)	13.71 (4.31)	0.959	1.041	4,98	0.390	0.041
B′	14.70 (5.91)	16.07 (5.79)						14.03 (5.73)	14.79 (5.00)						16.06 (6.09)	17.87 (6.44)					
C′	4.89 (2.47)	4.62 (2.07)						4.73 (2.38)	4.38 (2.16)						5.21 (2.63)	4.96 (1.92)					
D′	2.52 (1.18)	2.62 (1.49)						2.36 (1.18)	2.65 (1.37)						2.84 (1.13)	2.58 (1.67)					
Full Scale	33.70 (11.26)	35.26 (10.44)						32.31 (10.96)	32.53 (9.54)						36.53 (11.39)	39.13 (10.61)					

High IQ: IQ ≥ 85; Low IQ: IQ < 85; Domain A′, the domain of communication of ADI-R; Domain B′, the domain of social interaction abnormality of ADI-R; Domain C′, the domain of restricted and repetitive behaviors of ADI-R; Domain D′, the domain of exhibit specific aberrant behaviors that were present at or before the age of 36 months; MANCOVA, multivariate analysis of covariance (with age and FS-IQ as covariates).

**Table 7:** Sex differences among children with ASD in the domain scores of ADOS.

Domain	The total sample							High IQ							Low IQ						
	Male (*n* = 243) Mean (SD)	Female (*n* = 58) Mean (SD)	MANCOVA (Wilks’ Lambda)					Male (*n* = 163) Mean (SD)	Female (*n* = 34) Mean (SD)	MANCOVA (Wilks’ Lambda)					Male (*n* = 80) Mean (SD)	Female (*n* = 24) Mean (SD)	MANCOVA (Wilks’ Lambda)				
			*λ*	*F* value	*df*	*p* value	partial *η^2^*			*λ*	*F* value	*df*	*p* value	partial *η^2^*			*λ*	*F* value	*df*	*p* value	partial *η^2^*
A	3.18 (1.60)	3.03 (1.98)	0.970	3.019	3,295	0.030	0.030	2.77 (1.47)	2.41 (1.65)	0.984	1.018	3,191	0.386	0.016	4.01 (1.51)	3.92 (2.10)	0.878	4.555	3,98	0.005	0.122
B	7.44 (2.30)	6.79 (2.05)						6.88 (2.05)	6.44 (2.08)						8.56 (2.39)	7.29 (1.94)					
Full Scale	12.06 (4.06)	10.98 (4.21)						10.87 (3.44)	9.76 (3.67)						14.49 (4.16)	12.71 (4.39)					
C	Median (IQR)	Median (IQR)	Mann–Whitney *U* test					Median (IQR)	Median (IQR)	Mann–Whitney *U* test					Median (IQR)	Median (IQR)	Mann–Whitney *U* test				
			*Z* value		*p* value					*Z* value		*p* value					*Z* value		*p* value		
	1.00 (2.00)	1.00 (1.00)	−1.902		0.057			1.00 (2.00)	1.00 (1.00)	−1.653		0.098			2.00 (2.00)	1.00 (2.00)	−1.424		0.154		

High IQ: IQ ≥ 85; Low IQ: IQ < 85; Domain A, the domain of communication of ADOS; Domain B, the domain of reciprocal social interaction of ADOS; Domain C, the domain of restricted and repetitive behavior of ADOS; IQR, Interquartile range; MANCOVA: multivariate analysis of covariance (with age and FS-IQ as covariates).

## 4. Discussion

This study looks into possible sex differences in the core symptoms of children with ASD, as reported by parents and observed by testers. One point worth noting is that males with ASD have higher perceptual reasoning ability than females, despite having similar levels of overall intelligence. ADI-R did not find any statistically significant sex differences in the core symptoms of ASD. Significant effects of sex on ADOS were found in the reciprocal social interaction subcategory, especially among low-IQ children with ASD. Autistic children with low IQs are more likely to exhibit sex differences in their core symptoms than children with high IQs.

As is common knowledge, IQ and social interaction skills are strongly influenced by age and are highly variable and unstable in autistic preschoolers (28–30). Before the age of six, children’s brains are more plastic, and intervention is far more effective (31, 32). Indeed, decreases in autism symptom severity are more frequent in early childhood (17). Although early diagnosis and early intervention are significant themes within the field of autism research, the reality is that there is a widespread delay in the diagnosis of autism (33). The preschool years are critical for early intervention and early diagnosis, making them the primary focus of autism research. Because autism intervention is a long procedure, studying the clinical features of children with autism after the age of 6 years should not be overlooked. An in-depth investigation into the differences in core symptoms between boys and girls with autism will help to increase the diagnostic accuracy in clinical decision-making and individualization in the treatment and intervention processes. The majority of the various clinical assessments of autistic children will involve language development. Additionally, a child’s performance or score on an assessment in other domains will be impacted by their language proficiency. As a result, Wechsler IQ tests and other clinical assessments usually underestimate the abilities of autistic children who are nonverbal or have limited language skills, which makes it less likely that such individuals would measure reliably or validly (22, 28, 34). Standardized assessment tools, Wechsler IV, ADI-R, and ADOS-M3, were used for this study to examine the sex differences in clinical symptoms, allowing us to gain a deeper understanding of the clinical phenotypic traits of autistic children. The study’s focus was on school-age children and adolescents who were linguistically fluent.

Only in the area of perceptual reasoning did we find sex differences in the intellectual characteristics of linguistically fluent children with ASD. In line with earlier research, the Griffith evaluation revealed that boys with ASD had higher reasoning developmental quotients than girls (35). In addition, boys without ASD share the same perceptual reasoning advantage as boys with ASD (36). The variation in brain structure between males and females (37, 38) may aid in explicating the sex difference on the Perceptual Reasoning Index observed during our study. However, the association between perceptual reasoning ability and specific brain structures has yet to be confirmed by further research. The FS-IQ of children within the H-IQ group was considerably greater than that of the L-IQ group, and children within the H-IQ group were older than those in the L-IQ group. This result backs up the significant association between age and intelligence in children with ASD. The sex ratio (M/F) decreased among children with low IQ, and males had a higher FS-IQ than females in the low-IQ group, implying that female children with ASD are more likely to have comorbid intellectual impairment. Coinciding with a prior relevant study’s findings, boys are more likely than girls to have an IQ that is average or higher (39). This suggests that autistic girls could require additional interventional support addressing intellectual impairments.

The ADI-R assessment did not detect any statistically significant sex differences in all subcategory domain scores or diagnosis compliance rates. The conclusions remained consistent during the IQ-stratified analysis. In line with the results of earlier relevant investigations, the ADI-R and ADOS revealed no sex differences in the severity of ASD (8, 40). However, our finding was at odds with the result that indicated early-childhood RRB was lower in females (8). One possible reason for differences in results may be that our sample composition obscured any language- or intelligence-dependent sex differences in RRB in individuals with ASD. The small percentage of females in our sample is another explanation for the present results.

According to the findings of the ADOS test, sex differences in the core symptoms were found among the total sample, particularly in low-IQ ASD children. Females with low IQ were less severe than males in the reciprocal social interaction domain. Our results were not fully consistent with recent findings that ADOS-2 has no widespread systematic measurement bias based on race or sex (41). One possible and important reason may be that Kalb LG’s research did not consider the effect of intelligence on the results of ADOS. We observed sex differences in the core symptoms of ASD in children with low IQ but failed to find the difference in children with high IQ. This may be explained by the “camouflage mechanism.” Camouflaging was defined as the utilization of specific behavioral and cognitive strategies by autistic people to adapt to the predominately non-autistic social world (42–45). Emerging evidence suggests that autistic females demonstrate higher levels of camouflage than autistic males, and higher compensatory camouflage levels are linked to higher IQ (46, 47). Moreover, autistic males tend to exhibit more externalizing behaviors, while autistic females usually present more internalizing issues (48). As a result, it is more difficult for testers or clinicians to identify the behavioral manifestations of female autistic patients as autistic during the ADOS assessment. It is worth pondering that sex disparities in the core symptoms of autistic children may therefore be even more obvious if the study participants were further divided into subtle groups based on intelligence.

The ADI-R is a diagnostic instrument that evaluates children indirectly by using the descriptions of their parents or other primary caregivers. It is relatively subjective and usually subject to recall bias. Even with the aforementioned flaws or weaknesses, the ADI-R is a more comprehensive overview of the most aberrant states that the kid has experienced throughout his or her life and at the age of 4–5 years to reflect the traits of autistic children. ADOS ratings are based on a child’s immediate performance during testing, and evaluation results may be a little shaky or biased. ADI-R and ADOS can make up for each other’s deficiencies. At the overall level, the diagnostic compliance rate was analyzed for all subjects according to the diagnostic criteria for autism both in ADI-R and ADOS. There are three possible outcomes: the patients were identified as having “autism” by both ADI-R and ADOS, the patients were recognized as having “autism” based on only one of the two “gold standards,” and neither ADI-R nor ADOS were satisfied with the “autism” diagnosis. There were no sex differences in the composition ratios of the three conditions. Most individuals, whether males or females, met the autism diagnostic criteria in at least one of the diagnostic tools. While less than half of the children with fluent languages could satisfy the diagnostic criteria for both tests, what’s more, 15.64–17.24% of school-age children and adolescents with fluent languages will be underdiagnosed by both the ADI-R and the ADOS. It is worth noting that, even if an individual does not meet the diagnostic criteria for autism on either of the double A tests, it does not rule out the possibility that he or she has autism. Furthermore, a recent study reveals that developmental-behavioral pediatricians could diagnose ASD in young children without routinely using ADOS (49). When we performed stratified analysis according to cognitive level, we identified minor variations in core symptoms between boys and girls, but it was still difficult to discern differences in the performance of core symptoms between boys and girls with autism by double A alone. Children with average intelligence may use their cognitive abilities to cover up or make up for their social deficiencies. However, children in the low-IQ group may not notice much of a benefit from this compensating mechanism. This reinforces the hypothesis that sex differences might be more pronounced in autistic kids who have cognitive impairments. To thoroughly examine this assumption, a larger sample size is required as the majority of autistic children are moderate and do not have coexisting intellectual deficiencies.

Our study has several limitations that need to be addressed. Since the sample for this study was obtained from a single center, the research subjects might have been biased in their selection. Under the presumption of guaranteeing diagnostic homogeneity, multi-center collaboration could be employed in the future to enhance the sample’s representativeness. The study excluded nonverbal subjects, preschoolers, and individuals whose first language is not fluent, and the number of females and children with low IQ was small. Therefore, the findings from this study only reflect the tip of the iceberg for the autism spectrum community. In the future, in-depth analysis based on variables such as whether patients received intervention or not, the type of intervention, and the duration of the intervention will be necessary because the impact of intervention parameters on clinical symptoms was not taken into account in this study. The impacts of comorbidity on the clinical symptoms of autism were not taken into consideration in this research. Future research needs to be explored separately depending on whether the psychiatric comorbidity or the common comorbidity is present or absent in children with ASD, the type and severity of the comorbidity, and how they are managed or treated. In this study, one intelligence test tool and two reference standard diagnostic assessment tools were employed to measure sex differences in the symptoms of autism, and all these instruments were other-assessment tools. To better understand the sex differences of children with ASD, complementary information provided by self-assessment tests was necessary.

## 5. Conclusion

In general terms, overall cognitive functioning is similar across males and females with ASD; however, males have a higher perceptual reasoning ability. Female ASD is more likely to have comorbid intellectual impairment and could require additional intervention support. School-aged autistic children and adolescents with fluent language but low IQs are more likely to exhibit sex differences in their core symptoms than those with high IQs. Intelligence plays a key role in sex-based differences in the core symptoms of ASD. More representative populations and a thorough analysis of the underlying factors that may influence the clinical presentations are needed to deepen understanding of the differences between males and females with ASD.

## Data availability statement

The original contributions presented in the study are included in the article/supplementary material, further inquiries can be directed to the corresponding authors.

## Ethics statement

The studies involving human participants were reviewed and approved by the Ethics Committee of the Third Affiliated Hospital of Sun Yat-sen University. Written informed consent to participate in this study was provided by the participants’ legal guardian/next of kin.

## Author contributions

YaJ and YuJ: conceptualization, methodology, and writing—original draft. H-lZ and S-mC: data acquisition and curation, validation, and data analysis. X-bZ and F-lZ: conceptualization, project administration, supervision, and writing—review and editing. All authors contributed to the article and approved the submitted version.

## Funding

This study was supported by the National Natural Science Foundation of China (No. 81873801).

## Conflict of interest

The authors declare that the research was conducted in the absence of any commercial or financial relationships that could be construed as a potential conflict of interest.

## Publisher’s note

All claims expressed in this article are solely those of the authors and do not necessarily represent those of their affiliated organizations, or those of the publisher, the editors and the reviewers. Any product that may be evaluated in this article, or claim that may be made by its manufacturer, is not guaranteed or endorsed by the publisher.
